# Outpatient nursing support for self-monitoring in patients with chronic heart failure

**DOI:** 10.1371/journal.pone.0254019

**Published:** 2021-07-02

**Authors:** Chinatsu Taniguchi, Natsuko Seto, Yasuko Shimizu

**Affiliations:** 1 Division of Health Sciences, Graduate School of Medicine, Osaka University, Suita, Osaka, Japan; 2 Faculty of Nursing, Graduate School of Nursing, Kansai Medical University, Hirakata, Osaka, Japan; Universitat Luzern, SWITZERLAND

## Abstract

**Background:**

Early symptoms of worsening heart failure are difficult for patients to detect and manage, contributing to the high readmission rate for worsening heart failure. Thus, it is important to promote self-monitoring and to support patients in recognizing and interpreting their symptoms. This study aimed to explore the ways in which specialized nurses in the outpatient setting provide support for self-monitoring in patients with chronic heart failure in Japan.

**Methods:**

This exploratory study adopted a qualitative study design. The participants were a convenience sample of five nurses certified in chronic heart failure nursing and one advanced practice nurse certified in chronic care nursing, all with experience in outpatient nursing in Japan. Data were collected from June 2017 to October 2017 through semi-structured one-on-one interviews and were analyzed using an established qualitative inductive method.

**Results:**

The analysis identified seven themes describing the nursing support provided by the study participants. Among these were three themes describing different forms of direct support for self-monitoring: “Encourage patients to reflect on their own,” “Support touching the body and developing body awareness,” and “Support sharing the task.” Two themes described practice perspectives: “Support self-monitoring that is not overly sensitive” and “Support connection with the patient’s life.” Two final themes described contextual factors in the outpatient care setting: “Struggling with constraints and powerlessness” and “Building a support system in the outpatient setting.”

**Conclusions:**

The findings provide a practice for nurses promoting self-monitoring in patients with chronic heart failure in the outpatient setting. The study findings inform and provide goals for the support of self-monitoring in patients with heart failure and also, suggest the need to establish a strong support system for outpatient care in Japan.

## Introduction

Heart failure is a global problem affecting at least 26 million people worldwide [1], and its prevalence increases with age [2]. In the United States, the 60 to 90–day readmission rate for chronic heart failure is about 30% [1]. In Japan, its rehospitalization rate is 27% within 6 months and 35% within 1 year [3]. Thus, Japan has the same high readmission rate as the United States.

Cardiac function gradually declines with repeated acute exacerbations of chronic heart failure [4], but freedom from congestion has been shown to predict good survival [5]. Reducing delays in early diagnosis and the initiation of treatment for heart failure results in shorter hospital stays, shorter stays in the intensive care unit, lower mortality, and improved quality of life as well as in lowered resource use and health care costs [6,7]. Therefore, it is important for patients to recognize the signs of worsening heart failure early, to address their symptoms, and to have access to healthcare professionals.

Self-monitoring is awareness of symptoms or bodily sensations that is enhanced through periodic measurements, recordings, and observations to provide information for improved self-management [8]. Especially, self-monitoring in heart failure patients has three components, each requiring mastery: “awareness” (the subjective identification of the patient’s particular changing situation), “measurement” (the objective identification of the patient’s changing situation), and “interpretation” (the process of thinking about and attaching meaning to what one has been identified) [9]. Outcome studies have shown effective self-monitoring in heart failure patients to be associated with decreased mortality, heart failure decompensation events, emergency visits, hospitalizations, length of stay, and costs and with increased weight awareness and symptom awareness and increased contact with healthcare professionals [10]. Therefore, it is expected that support for self-monitoring will lead to early detection of worsening heart failure and the initiation of appropriate measures and will contribute positively to the medical economy.

Despite the importance of early symptom recognition, the mismatch between objective and subjective detection of fluid retention has been reported [11], and it is known that it is difficult for patients to identify early symptoms. Patients are advised to weigh themselves daily, but it is difficult to accurately distinguish between an increase in fluid volume versus fat [12]. Finally, it is difficult to identify the signs of exacerbation based solely on the presence or absence of symptoms or on measures such as blood pressure [9]. Therefore, it is necessary to support patients in their efforts to recognize and interpret symptoms.

Various intervention studies have investigated self-monitoring in patients with chronic heart failure [13–17]. Many of the studied interventions aimed at improving symptom detection through improved knowledge and self-monitoring skills. This has been a recognized need—following discharge, patients must engage in daily self-care, but learning to manage heart failure independently is challenging for many [18]. Thus, it is important that patients have the necessary knowledge and skills and also, support as they assume this new challenge. Despite this, it is unclear how nurses provide support for patients facing difficulties with self-monitoring.

We considered that investigating the practices of specialist and advanced practice nurses in the outpatient setting would provide valuable insight into supportive care for patients with chronic heart failure. The purpose of this study was to explore how nurses in the outpatient setting in Japan provide advanced support for self-monitoring to patients with chronic heart failure.

## Methods

### Participants

The study participants were nurses with experience providing outpatient support for patients with chronic heart failure and were either advanced practice nurses specializing in chronic care nursing with chronic heart failure nursing as a subspecialty or were nurses certified in chronic heart failure nursing. Thus, all participants had completed either a specific master’s program or six months of specialty training, in addition to a minimum of three years of work experience in the specialty.

Convenience sampling was used to recruit the participants: most were recruited through a professional network of nurses certified for chronic heart failure nursing; additional participants were known to the authors and were invited to participate based on their superior communication skills. The participants worked in outpatient departments of hospitals in the Kansai, Chugoku, and Tohoku regions of Japan, and none worked at the same facility.

### Data collection

The data were collected though semi-structured one-on-one interviews (approximately 60 minutes each) from June 2017 to October 2017. The interviews included the questions: How do you provide support for self-monitoring to outpatients with chronic heart failure, and what kinds of support are effective? If a patient has difficulty with self-monitoring, how do you help? Additionally, the interviewer collected data on the services provided by the respective nurses, to better understand feasible means of support. For consistency, all interviews were conducted by the lead author (CT). All interviews were conducted in private at a mutually convenient location, and with the consent of the participants, all the interviews were recorded and transcribed.

### Data analysis

The qualitative synthesis method developed by Jiro Kawakita (the KJ method) was used to analyze the data [19–22]. The KJ method uses qualitative inductive analysis to extract meaning and essence from a random situation and permits the structural expression of data, without abstracting the many elements found in the phenomenon.

The KJ method involves three steps of analysis: label making, grouping, and chart making [19–22]. During label making, data extracted from the interview transcripts were classified such that each piece of semantic content was assigned a label. During this step, care was taken to preserve the integrity of participants’ statements. In the second step, the created labels were laid out for easy readability. Then, the labels were read through repeatedly, and labels with similar semantic content were grouped onto two to three sheets. After the initial grouping, the essence of each of the grouped labels was expressed in a short summary, and the grouping exercise was repeated with the generated short summaries used as the label units. Grouping was repeated in this way until five to seven sheets of label units had been produced. Finally, in the third step, the final labels were spatially arranged and a chart constructed. In constructing the arrangement, the logical relationships between the labels were explored and the resulting structure visualized.

In this study, focusing on how specialized nurses supported self-monitoring in chronic heart failure patients, the verbatim transcripts generated in the interviews were used as data. As a result, a total of 511 labels were initially created. It was difficult to analyze them all at once due to the large number of labels. Therefore, we followed the general process, but did not analyze the coded labels together. Instead, we assigned random numbers to each label, randomly divided these in half, and then grouped the labels in each half until the number of labels was less than 150. After that, all the labels were merged and we obtained 267 labels. These labels were analyzed as usual.

### Credibility and authenticity

The analysis was performed by two researchers, CT (who completed KJ method instructor training) and YS (a qualified KJ method instructor). The two researchers discussed the entire analysis process, and decisions at every step were based on consensus.

### Ethical considerations

This study was approved by the Mukogawa Women’s College Ethics Review Board (No. 16–80). The research aims and procedures were explained, verbally and in writing, to all candidate participants when their consent for participation was sought. Participants were informed that participation was voluntary, that they had the option of refusing to participate or of withdrawing at any time, that the anonymity of individual participants would be preserved, that the study findings could be disclosed, and that the researchers would store all data under conditions of strict confidentiality and dispose of the data at the conclusion of the study. All participants provided written consent.

## Results

A total of six nurses participated in the study, of whom five were certified in cardiac care nursing and one was certified as an advanced practice nurse in chronic care nursing. The nurses (5 females, 1 male) ranged in age from 36 to 45 years (mean 40.8 years). Further details of the sample are shown in Table 1. The interview times averaged 59.8 minutes.

**Table 1:** Details of the study participants.

Participant	General nursing experience (years)	Cardiovascular nursing experience (years)	Certified nursing experience (years)
A	14	14	5
B	13	10	5
C	24	19	4
D	25	25	6
E	21	21	4
F	17	17	2

**Fig 1:**
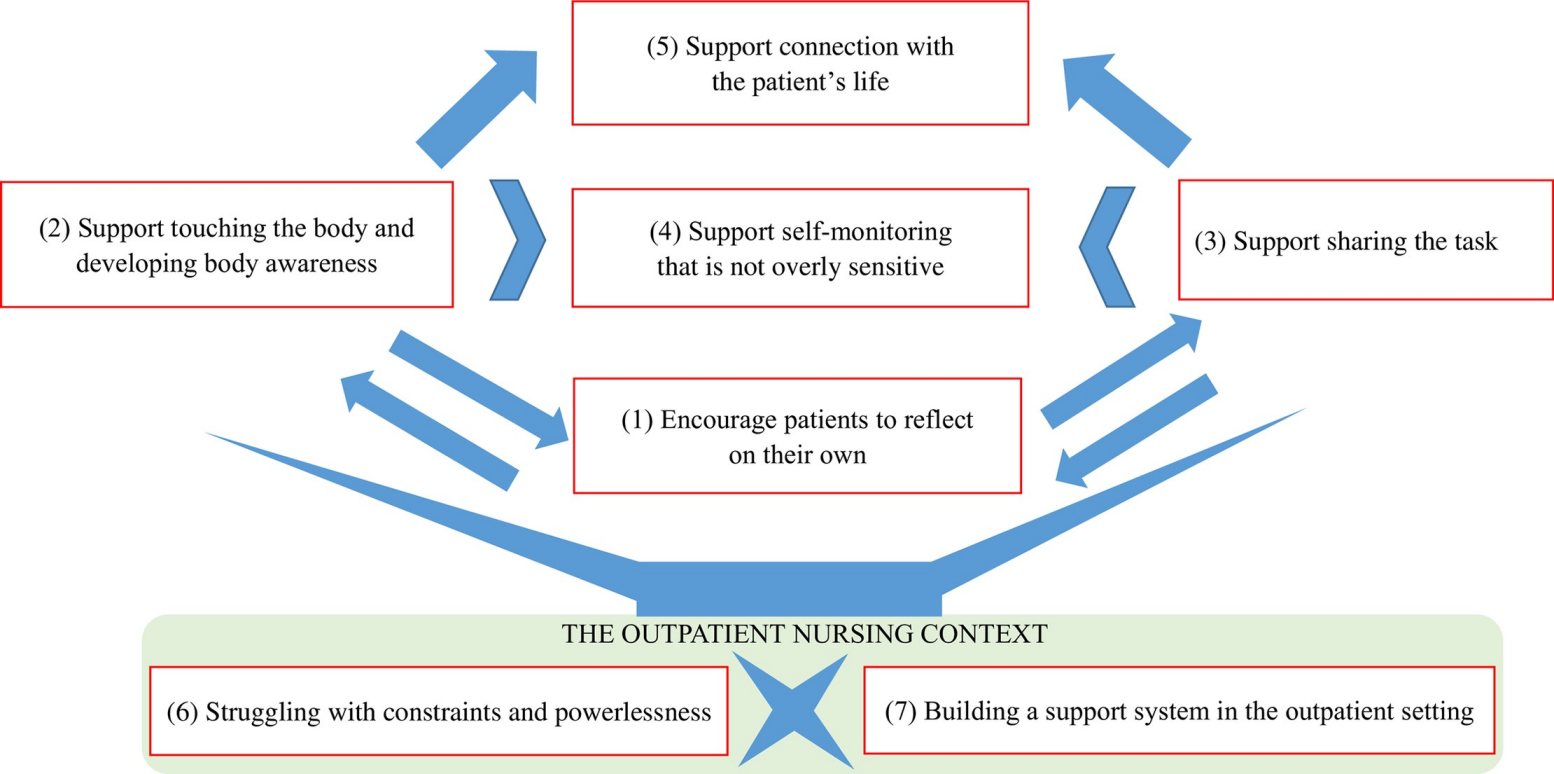
Support and the outpatient care context, for self-monitoring in patients with chronic heart failure. Note: The arrows indicate the direction of influence. The chevrons indicate situational influences. The cross indicates conflict.

Seven themes were identified in the analysis. These included five main themes describing the support provided for patient self-monitoring: “Encourage patients to reflect on their own,” “Support touching the body and developing body awareness,” “Support sharing the task,” “Support self-monitoring that is not overly sensitive,” and “Support connection with the patient’s life.” Two additional themes described contextual factors in the provision of support: “Struggling with constraints and powerlessness” and “Building a support system in the outpatient setting.” The relationship between the themes is shown in Fig 1.

### (1) Encourage patients to reflect on their own

The theme “Encourage patients to reflect on their own” captured the nurses’ aim of reinforcing patient efforts to self-monitor. The nurses reported that until the patient becomes skilled at the process of self-monitoring (including recording and interpreting the physical condition), they attempt to encourage the patient’s efforts, and nursing actions aim to foster patient reflection, awareness, and ability to voice concerns; At the same time, the nurses encourage patients to engage in self-care. As such the nurses attempt to motivate the patient to continue with self-monitoring efforts, while determining whether there are signs of heart failure exacerbation.

*“I started with the story, ‘You have been hospitalized many times and often looked at [the oxygen values]’ and said, ‘The oxygen [level] was higher when you felt comfortable.’ To the [patient’s] point that ‘it makes me breathless when water accumulates,’ I asked ‘Did you put up with this discomfort at home?’ …[I encouraged the patient to] go back in time and bring this back to the current situation and difficulties…. to make comparisons with situations when the patient was feeling well at home” (SG44)*

### (2) Support touching the body and developing body awareness

The second theme described nurses’ efforts to promote body exploration and body awareness. Nurses reported believing the patient’s own experience and meanings to be as important as the objective data. Thus, cultivating patient body awareness and exploration was important for them. The nurses reported touching the patient’s hands and feet to reflect where and how symptoms and signs are found, encouraging the patient to “try this out on your body.” This focuses patient awareness on the body and helps equip the patient to identify symptoms and act on concerns.

*“I often check the patient’s physical condition by feeling for coldness in the periphery, so I always touch the patient’s hand, while asking ‘May I touch [you]?’ Most patients will reach out when I [touch particular places] and say, ‘Today, it’s warm [here].’ That happens in almost all cases. Then, when the patient is not feeling well, he might say, ‘I have cold hands.’ That’s why I think this is also a part of self-monitoring.” (W-010)*

### (3) Support sharing the task

The third theme captured nurses’ recognition of the need, in some cases, to enlist the help of others to share the task of self-monitoring. Some patients have difficulty measuring or recording their weight or have difficulty interpreting whether contact with a medical professional is indicated; in these cases, nurses reported asking family members or visiting nurses to “check in” (in person or by phone) with the patient on the difficult part. Similarly, nurses may request that the patient return more often the outpatient clinic for minor adjustments.

*“The purpose is to get an early consultation [as soon as this is needed]. It is quite possible for patients over 80 years old to weigh [themselves] and write down [the weight], but it is a little difficult to decide whether to go to the clinic when they reach [a certain] weight. In the case of one older couple, the patient weighed himself regularly, but he should have contacted a medical professional when he was about this weight…. When patients are hospitalized, I sometimes think about this when I look at their notebook [for self-monitoring], so I wonder if it’s okay [to call two weeks after discharge to check up on their condition and get them an early consultation if necessary].” (K31)*

### (4) Support self-monitoring that is not overly sensitive

The fourth theme captured the nurses’ perspective that for some patients, involvement in self-monitoring could be taken to a harmful extreme. The nurses reported instances where self-monitoring increased anxiety in patients or where patients were inappropriately swayed by the numerical values. These included cases where the patient was overly affected by elevated blood pressure values, where the patient self-monitored so frequently and was so body sensitive that this interfered with sleep, and where the patient recorded detailed daily findings but felt subjectively better than the numerical values showed. Nurses reported thinking they ought to stop patients who measure “too much” and that they are sometimes reticent to share test results with an anxious patient.

*“In some cases, I failed because I strengthened the patient’s self-monitoring too much …. the patient became overly sensitive to his body and started to worry about the little things. That’s right. One patient who was really good at self-monitoring was well enough for [activities like] golfing despite his poor heart function, but he became depressed, and I got a phone call. He was so worried that he couldn’t sleep at night and didn’t know what to do. I think it’s very difficult [when this happens].” (W-027)*

### (5) Support connection with the patient’s life

The theme “Support connection with the patient’s life” captures the nurses’ patient-centered perspective that successful self-monitoring is based on the patient’s engagement and acceptance of the ongoing task. The nurses aimed to create in the patient the sense that “I get it,” by drawing lines and connecting the medical task in ways that were meaningful to the patient and aimed to allow patients to decide for themselves how to fit self-monitoring in their lives. The nurses also recognized the importance of creating “space,” i.e., room for patients to enjoy leisure time and activities when feeling well.

*“When the patient can eventually begin self-monitoring, I want [the patient] to connect with it, so that the patient’s senses are sharp and the reasoning is understood. So, besides the patient’s own action of contacting the medical staff, I think the most important thing is to create space where [the patient] can rest, eat, and, on the contrary, enjoy a little leisure time.” (W-072)*

### (6) Struggling with constraints and powerlessness

The sixth theme described different challenges that are unique to the outpatient nursing setting. Nurses everywhere play multiple roles and face numerous challenges; nevertheless, the participants struggled with the role of a certified nurse in the outpatient setting because of the lack of time for reflection. The nurses also believed that nurses in advanced roles need to better highlight the importance of the outpatient nursing role in the interprofessional team; what else should be done patients in situations where recuperation is difficult, before initiation of self-monitoring.

*“In a university hospital, when extra work comes in, I wonder if I am really doing my job properly. But right now, somehow, I’m already doing … coordination—a coordinating role for the other professionals. And I’m hoping that I can give back to the patients by doing ‘behind the scenes’ stuff there.” (SN97)*

### (7) Building a support system in the outpatient setting

The final theme involved nursing activities related to outpatient setting processes, including communication and referral processes. The nurses recognized the need for good communication to ensure involvement of the full medical team and reported their sharing of results from the patient handbook (the self-monitoring record), use of teaching checklists, and support for consultation. Other activities included initiatives for improving community partnerships, referral suggestions, and workshops publicizing the important roles of outpatient nurses.

[*Replying to the question*: *So*, *you*’*re saying that all* patients *are given a heart failure handbook*?*]*

*“Yes, that’s right. We were told to give [the handbook to] almost all patients diagnosed with heart failure at our hospital, so we first created a project team like this in the hospital, and then we created networks—between the outpatient clinic and the Coronary Care Unit, and the wards and the outpatient clinic—and that’s how we first introduced it, and that’s how it’s continued.” (SN14)*

## Discussion

This study clarified the structure of the support offered by specialist nurses to promote self-monitoring in outpatients with chronic heart failure. Five forms of support for self-monitoring emerged; among these, are three core forms of support, “Encourage patients to reflect on their own,” “Support touching the body and developing body awareness,” and “Support sharing the task”; two additional perspectives on support were also described, “Support self-monitoring that is not overly sensitive” and “Support connection with the patient’s life” (the ultimate goal of self-monitoring). In addition, it became clear that these are practiced in (and influenced by) the context of the outpatient nursing setting, described by the two themes of “Struggling with constraints and powerlessness” and “Building a support system in the outpatient setting.”

First, it was described that an attitude of involvement and acceptance of the patient’s reflection and perspective provides the foundation for facilitating patient self-monitoring. The nurses took care not to negate the patient experience and instead, created an atmosphere in which it was easy for the patient to talk and asked deliberate questions to stimulate patient reflections about symptoms, behaviors, and thoughts. An important factor in symptom interpretation is the allowance of time to think through the symptom experience [23]. The acts of listening to patient perceptions and experiences and introducing the physical condition and parameter values in a manner that is meaningful for the patient will promote reflection and heightened awareness.

Next, nurses “Encourage patients to reflect on their own” and at the same time, also engage in “Support touching the body and developing body awareness.” The nurses expressly touched areas that patients needed to be aware of, such their hands, demonstrating and explaining how to check for peripheral circulation and edema, to enable their patients to recognize the relationship between the signs of heart failure and their physical condition. This is both a simple and potentially effective way to facilitate patient awareness of body changes without the need for special technology. Similarly, in previous work Yoneda reported on the development of a Body Sensation Care Model for patients with type 2 Diabetes [24], which was intended to promote body awareness. It was suggested that this intervention, like the interventions of nurses in the present study, helped patients to become aware of and capture body sensations and provided meaningful support. According to an integrative review of symptom perception studies by Lee et al. [25], it was recommended for considering interventions for self-assessment of edema to improve the recognition and interpretation of heart failure symptoms. In addition, they reported interventions that aid in body listening is useful in clinical practice, and the intervention in this study may be helpful.

The nurses also “Support sharing the task.” Skill in self-monitoring is acquired gradually, through experience [23]. In some cases, cognitive problems related to heart failure and aging [23] may slow the recognition and interpretation of symptoms. Until the patient can perceive and interpret symptoms independently, it may be necessary to enlist the help of others to facilitate self-monitoring. The nurses in this study recognized that the task of self-monitoring does not have to be the sole responsibility of the patient. Rather than labeling patients as “incapable,” it is important to support patients in taking decisions about this, and it may be appropriate to have them do the part they can do themselves and to support sharing the remaining part with others.

An important perspective revealed in this study was that for some patients, it is preferable to provide support for less attentiveness in self-monitoring. In part, this may be related to the high mortality rate with heart failure [1,26] and resulting high prevalence of depression and anxiety in patients with heart failure—in Japan, the prevalence of depression and anxiety is 37% for each [27], which suggests that depression and anxiety are already common in outpatients with heart failure. Further, brain natriuretic peptides above 200 ng/L are associated with heightened anxiety [27], suggesting that the severity of heart failure may affect the level of anxiety. While self-monitoring will help patients to develop body awareness and better understand their symptoms, self-monitoring may also have paradoxical negative psychological effects. It is necessary for nurses to evaluate this and to provide careful support to patients with particularly high anxiety. Previous studies have explored interventions to promote self-monitoring. However, to the best of our knowledge, we have not found any studies that have discussed the negative aspects of self-monitoring. In the future, it will be necessary to examine the psychological impact of self-monitoring.

The ultimate goal in promoting self-monitoring is to integrate self-monitoring into the patient’s lifestyle. Thus, we found self-monitoring support that aimed to create as much free space in the patient’s life as possible. In providing self-monitoring support to patients with chronic heart failure, it is easy to ignore the fit of this in the patient’s life; however, while it is important to support patients in recognizing their own bodies and interpreting their physical condition appropriately, it is equally important to provide support for their life choices.

The nurses provided their support for outpatient self-monitoring within the context of the challenges and professional support system in the outpatient setting. Outpatient practice is complex, and the nurses reported struggling with their advanced role and their approach to patients. In the outpatient setting, there is need for nurses to organize their work and to create an environment that facilitates professional consultation. At the same time, we also found nurses working to optimize the outpatient support system—forming teams, communicating with other medical staff and the community, and using tools such as the Heart Failure Handbook in support of patient self-monitoring. In Japan, it will be important to establish a national system that will enable the entire medical community to provide such support.

The study participants were a certified heart failure nurse and chronic disease nurses in Japan. While the rich experience of specialized and advanced practice nurses is informative and useful, we recognize that a limited number of participants were surveyed; Further study, to capture more diverse support content, is indicated. In addition, studies are needed to verify the effectiveness of specific interventions based on the support identified in this study and to accumulate evidence for the support methods.

## Conclusions

This study clarified the structure of the support offered by specialist nurses to promote self-monitoring in outpatients with chronic heart failure by using an established qualitative inductive method. As a result, five themes of support for self-monitoring and two themes of the context of the outpatient nursing setting emerged. The findings inform and provide goals for the support of self-monitoring in patients with chronic heart failure. Furthermore, it was shown that self-monitoring may have paradoxical negative psychological effects. Therefore, it is necessary for nurses to evaluate this and to provide careful support to patients with particularly high anxiety. As future work, further research is needed to evaluate the effectiveness of specific nurse interventions, and there is also a need to establish a strong support system for outpatient care in Japan.
